# Prognostic values of DNA mismatch repair genes in ovarian cancer patients treated with platinum-based chemotherapy

**DOI:** 10.1007/s00404-017-4563-x

**Published:** 2017-10-23

**Authors:** Chuchu Zhao, Saisai Li, Menghuang Zhao, Haiyan Zhu, Xueqiong Zhu

**Affiliations:** Department of Obstetrics and Gynecology, The Second Affiliated Hospital of Wenzhou Medical University, No. 109 Xueyuan Xi Road, Wenzhou, 325027 Zhejiang China

**Keywords:** Mismatch repair, Ovarian cancer, Prognosis, KM plotter

## Abstract

**Purpose:**

DNA mismatch repair (MMR) is a highly conserved biological pathway that plays a key role in maintaining genomic stability. MMR has been reported as a prognostic marker in certain cancers; however, the results are controversial. Therefore, identification of the prognostic value of MMR genes in ovarian cancer based on a large sample size is pivotal.

**Methods:**

In the current study, we systemically investigated the prognostic roles of seven MMR genes, MSH2, MSH3, MSH6, MLH1, MLH3, PMS1 and PMS2, in ovarian cancer patients treated with platinum-based chemotherapy through “The Kaplan–Meier plotter” (KM plotter) database, which contains gene expression data and survival information of ovarian cancer patients.

**Results:**

Among seven MMR genes, high mRNA levels of MSH6, MLH1 and PMS2 were significantly associated with a better overall survival for all ovarian cancer patients treated with platinum-based chemotherapy, especially in late-stage and poor-differentiated ovarian cancer patients. Increased MSH6 and PMS2 mRNA expression was correlated with a favorable overall survival in serous ovarian cancer patients.

**Conclusions:**

Our results indicate that sufficient MMR system is associated with an improved survival in ovarian cancer treated with platinum-based chemotherapy. MMR gene may be a potential prognosis predictor in ovarian cancer.

## Introduction

Approximately 238,700 new cases of ovarian cancer have been diagnosed worldwide, with estimated 151,900 associated deaths in 2012 [1]. The high mortality rate of ovarian cancer is primarily due to late detection, drug resistance and deficiency target therapy [2, 3]. Although patients are initially sensitive to conventional chemotherapy following debulking surgery, most of them experience recurrence within 12–24 months and ultimately die of the disease. Consequently, identification of potential prognostic biomarkers and development of novel therapeutic strategies in ovarian cancer are urgently needed to improve clinical outcome.

Mismatch repair (MMR), an evolutionarily conserved mechanism that corrects mutations arising during DNA replication or damage, plays a crucial role in maintaining genome stability [4–6]. MMR system is a multi-step process involving key components at each stage. Seven MMR genes, mutL homolog 1 (MLH1), mutL homolog 3 (MLH3), mutS homolog 2 (MSH2), mutS homolog 3 (MSH3), mutS homolog 6 (MSH6), postmeiotic segregation increased 1 (PMS1), postmeiotic segregation increased 1 (PMS2) are involved in human MMR function [5, 7]. It is now well-known that inactivation of MMR in human cells is associated with genome-wide instability, including microsatellite or DNA damage, predisposition to certain types of cancer [8–13]. In ovarian cancer, MMR deficiency is the most common cause of hereditary ovarian cancer after BRCA1 and BRCA2 mutations [7].

The potential of MMR system as a prognostic predictor has been intensely evaluated and has shown great promise in certain cancer types, especially in colorectal cancers and endometrial cancers. Patients with deficient MMR colorectal or colon tumors commonly have an improved survival rates compared with patients with proficient MMR cancers [14, 15]. Inversely, inactivation of MMR in endometrial cancer is correlated with negative prognostic factors and worse progression-free survival [16, 17]. With respect to ovarian cancer, few studies have investigated the prognostic significance of MMR defects and with inconsistent results [18–20]. Moreover, none of these studies have systematically evaluated the prognostic value of individual MMR component, especially in the mRNA level, in ovarian cancer. In the current study, we accessed the prognostic value of individual MMR component among 1335 human ovarian cancer patients treated with platinum-based chemotherapy using an online Kaplan–Meier plotter (KM plotter), which integrates gene expression and clinical data including survival information on 1648 ovarian cancer patients [21].

## Materials and methods

An online database (http://kmplot.com/analysis/) was used to assess the prognostic value of individual MMR genes mRNA expression among ovarian cancer patients. This database integrates gene expression and clinical data and is capable to evaluate the effect of 54,675 genes on survival using 10,188 cancer samples, including 1648 ovarian cancer samples with a mean follow-up of 40 months. Gene expression data and progression-free and overall survival information in this database are downloaded from Gene Expression Omnibus (GEO), EGA and the Cancer Genome Atlas (TCGA) [21]. To analyze the prognostic value of a particular gene, the patient samples are split into two groups according to various quantile expressions of the proposed biomarker. MMR expression status were finally classified into “low” and “high” according to the comparisons between expression values with established cut-offs. The two patient cohorts are compared by a Kaplan–Meier survival plot, and the hazard ratio (HR) with 95% confidence intervals (CI) and log-rank *P* value are calculated.

Currently, this database (2015 version) has already collected clinical data including progression-free and overall survival information, stage, histology, grade, TP53 mutation, debulk and treatment of ovarian cancer patients [21]. A summary of the clinical characteristics of the patients used in the analysis is shown in Table 1. Shortly, seven MMR members (MSH2, MSH3, MSH6, MLH1, MLH3, PMS1 and PMS2) were entered into the database (http://kmplot.com/analysis/index.php?p=service&cancer=ovar) to obtain Kaplan–Meier survival plots. *P* value of < 0.05 was considered to be statistically significant.

**Table 1:** Clinical characteristics of ovarian cancer patients

Variable	*N*
Clinical stage	
I + II	91
III + IV	1027
Pathological grade	
I	30
II	285
III	816
Histological subtype	
Serous	971
Endometrioid	30
TP53 mutation	
Yes	402
No	82
Debulk	
Optimal	716
Suboptimal	454
Death event	724
Median OS	32.43 (m)

*N* number of patients with available clinical data, *OS* overall survival, *m* months

## Results

There are seven MMR genes involved in human MMR function: MLH1, MLH3, MSH2, MSH3, MSH6, PMS1 and PMS2. All of these seven MMR genes’ survival information can be found in http://www.kmplot.com. Survival curves were plotted in http://www.kmplot.com for 1335 human ovarian cancer patients treated with platinum-based chemotherapy (follow-up time 20 years), including 971 serous ovarian cancer patients and 30 endometrioid ovarian cancer patients. The desired Affymetrix IDs is valid: 209421_at (MSH2), 205887_x_at (MSH3), 202911_at (MSH6), 202520_s_at (MLH1), 204838_s_at (MLH3), 213677_s_at (PMS1) and 221206_at (PMS2).

**Fig. 1:**
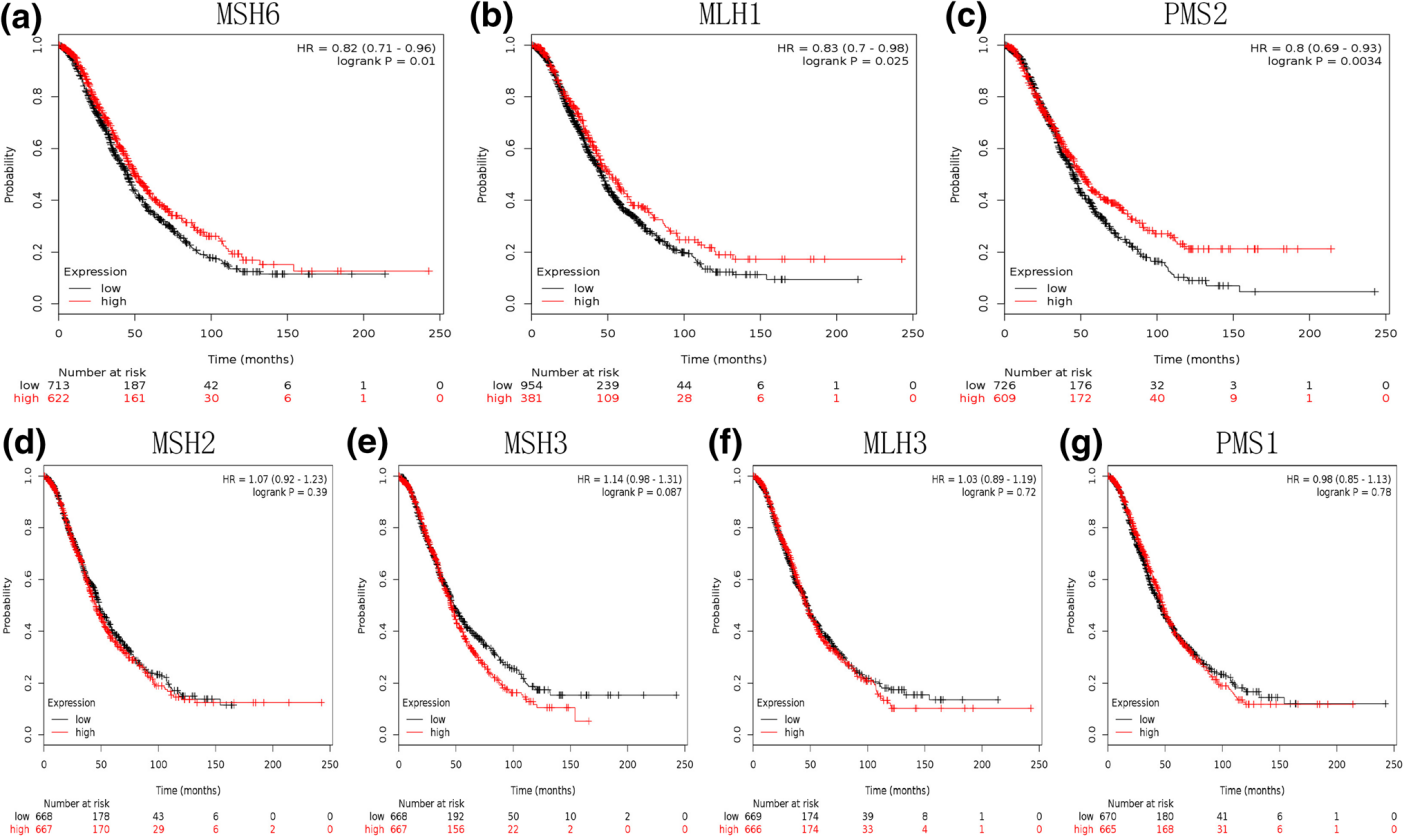
Determination of prognostic value of seven MMR genes mRNA expression in 1335 human ovarian cancer patients treated with platinum-based chemotherapy. **a** Survival curves are plotted for MSH6. **b** Survival curves are plotted for MLH1. **c** Survival curves are plotted for PMS2. **d** Survival curves are plotted for MSH2. **e** Survival curves are plotted for MSH3. **f** Survival curves are plotted for MLH3. **g** Survival curves are plotted for PMS1

When the whole patients’ population was analyzed, high mRNA expression of MSH6 (HR 0.82, 95% CI 0.71–0.96, *P* = 0.01), MLH1 (HR 0.83, 95% CI 0.7–0.98, *P* = 0.025), and PMS2 (HR 0.8, 95% CI 0.69–0.93, *P* = 0.0034) was associated with a significantly improved OS, whereas the mRNA expression of MSH2, MSH3, MLH3 and PMS1 genes was not related to OS of ovarian cancer treated with platinum-based chemotherapy (Fig. 1).

**Fig. 2:**
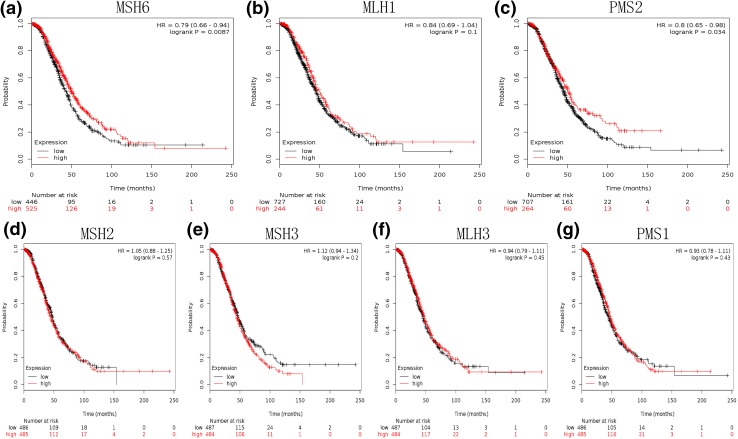
Determination of prognostic value of seven MMR genes mRNA expression in 971 serous ovarian cancer patients treated with platinum-based chemotherapy. **a** Survival curves are plotted for MSH6. **b** Survival curves are plotted for MLH1. **c** Survival curves are plotted for PMS2. **d** Survival curves are plotted for MSH2. **e** Survival curves are plotted for MSH3. **f** Survival curves are plotted for MLH3. **g** Survival curves are plotted for PMS1

**Fig. 3:**
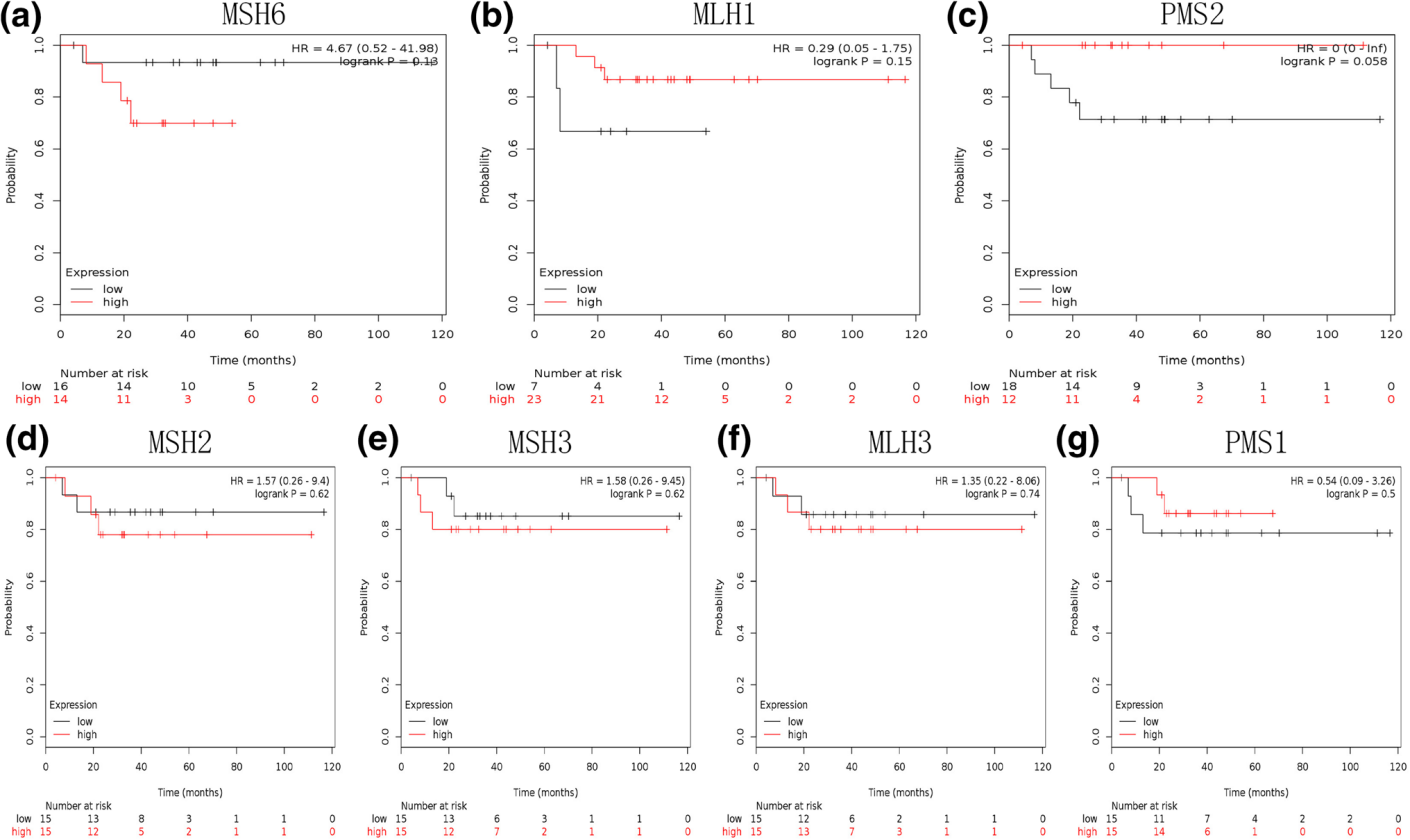
Determination of prognostic value of seven MMR genes mRNA expression in 30 endometrioid ovarian cancer patients treated with platinum-based chemotherapy. **a** Survival curves are plotted for MSH6. **b** Survival curves are plotted for MLH1. **c** Survival curves are plotted for PMS2. **d** Survival curves are plotted for MSH2. **e** Survival curves are plotted for MSH3. **f** Survival curves are plotted for MLH3. **g** Survival curves are plotted for PMS1

Considering risk factors, molecular events, prognostic markers, and therapeutic targets vary substantially across subtype in epithelial ovarian cancer. We then evaluated the prognostic significance of MMR genes in serous and endometrial ovarian cancer, respectively. High mRNA expression of MSH6 (HR 0.79, 95% CI 0.66–0.94, *P* = 0.0087) and PMS2 (HR 0.8, 95% CI 0.65–0.98, *P* = 0.034) was correlated to a favorable OS in serous cancer patients (Fig. 2). However, none of the MMR genes was significantly associate with OS in endometrial ovarian cancer (Fig. 3).

We further accessed the correlation between MMR genes and OS of ovarian cancer in different clinicopathological features ovarian cancer patients. We determined the correlation with clinical stages (Table 2), pathological grades (Table 3) and TP53 mutation (Table 4) in ovarian cancer patients. As shown in Table 2, increased MSH6 and MLH1 mRNA levels were associated with a better OS in stage III + IV ovarian cancer patients, high mRNA levels of PMS2 implied an improved OS either in stage I + II or stage III + IV ovarian cancer patients. As shown in Table 3, while high levels of MLH1 were associated with a better OS in grade III ovarian cancers and increased mRNA expression of PMS2 was positively correlated with OS in grade II ovarian cancer, MSH6 mRNA expression was a favorable predictor of OS both in grade II and grade III ovarian cancer patients. As shown in Table 4, over-expression of PMS2 was correlated to a worse OS in TP53 wild-type ovarian cancer patients, HR 2.15 (1.06–4.36), *P* = 0.03. None of the MMR genes was associated with OS in TP53 mutated ovarian cancer patients.

**Table 2:** Correlation of MMR gene expression level with overall survival in ovarian cancer patients with different clinical stage

MMR genes	Clinical stages	Cases	HR	95% CI	*P* value
MSH2	I + II	91	1.52	0.58–4.01	0.39
	III + IV	1027	0.95	0.81–1.12	0.56
MSH3	I + II	91	1.53	0.58–4.03	0.39
	III + IV	1027	1.08	0.92–1.28	0.34
MSH6	I + II	91	1.93	0.63–5.91	0.24
	III + IV	1027	0.79	0.67–0.93	0.0051*
MLH1	I + II	91	0.46	0.17–1.21	0.11
	III + IV	1027	0.77	0.64–0.93	0.0065*
MLH3	I + II	91	0.78	0.3–2.04	0.62
	III + IV	1027	1.03	0.87–1.21	0.74
PMS1	I + II	91	1.18	0.45–3.11	0.74
	III + IV	1027	1	0.84–1.18	0.97
PMS2	I + II	91	0.36	0.14–0.95	0.032*
	III + IV	1027	0.74	0.61–0.88	0.0009*

*P < 0.05

**Table 3:** Correlation of MMR gene expression level with overall survival in ovarian cancer patients with different pathological grade

MMR genes	Pathological grades	Cases	HR	95% CI	*P* value
MSH2	I	30	1.24	0.37–4.11	0.72
	II	285	1.06	0.77–1.47	0.71
	III	816	0.99	0.82–1.2	0.95
MSH3	I	30	4.22	1.1–16.12	0.023*
	II	285	1.15	0.83–1.59	0.4
	III	816	1.07	0.88–1.29	0.49
MSH6	I	30	4.76	1.24–18.19	0.013*
	II	285	0.7	0.5–1	0.046*
	III	816	0.74	0.61–0.9	0.0021*
MLH1	I	30	0.48	0.12–1.82	0.27
	II	285	0.68	0.46–1.01	0.055
	III	816	0.73	0.58–0.91	0.0056*
MLH3	I	30	0.9	0.27–2.96	0.86
	II	285	1.08	0.78–1.49	0.66
	III	816	0.93	0.77–1.12	0.42
PMS1	I	30	1.05	0.32–3.44	0.94
	II	285	0.92	0.66–1.27	0.6
	III	816	0.96	0.79–1.16	0.64
PMS2	I	30	0.41	0.09–1.92	0.24
	II	285	0.63	0.45–0.88	0.0071*
	III	816	0.84	0.67–1.04	0.1

*P < 0.05

**Table 4:** Correlation of MMR gene expression level with overall survival in ovarian cancer patients with different TP53 mutation status

MMR genes	TP53 mutation	Cases	HR	95% CI	*P* value
MSH2	Yes	402	1.13	0.86–1.47	0.39
	No	82	0.75	0.41–1.37	0.35
MSH3	Yes	402	1.01	0.78–1.32	0.93
	No	82	1.13	0.62–2.06	0.7
MSH6	Yes	402	0.78	0.59–1.01	0.063
	No	82	1.73	0.85–3.52	0.13
MLH1	Yes	402	0.86	0.65–1.14	0.3
	No	82	1.54	0.85–2.79	0.15
MLH3	Yes	402	0.87	0.67–1.14	0.31
	No	82	1.22	0.68–2.21	0.51
PMS1	Yes	402	0.9	0.69–1.18	0.44
	No	82	0.91	0.5–1.63	0.74
PMS2	Yes	402	0.82	0.63–1.08	0.15
	No	82	2.15	1.06–4.36	0.03*

*P < 0.05

Expect for the results mentioned above, the mRNA expression of MSH2, MSH3, MLH3 and PMS1 genes were not associated with OS in different clinical stages and different grades ovarian cancer patients.

## Discussion

The occurrence and tumorigenesis of cancer is a complicated multi-step process, which involves numerous factors, including multiple gene mutations. MMR genes have been thought to be crucial to the occurrence, development and clinicopathological features of cancer. When the MMR system develops a functional error or defect, the repair process fails and unrepaired mutations become scattered throughout the genome, resulting in mutations in cancer-related genes [22]. Indeed, a disrupted MMR system has been identified in several types of cancers including gastric cancer [23], endometrial carcinoma [13], colorectal cancer [24, 25], breast cancer [26, 27], pancreatic cancer [28, 29], prostate cancer [30] and Wilms tumor [31]. Furthermore, MMR has been reported as a prognostic marker in certain cancers; however, the results are controversial.

In the presented study which, in the best of our knowledge, is the first work, we systematically analyzed the prognostic value of seven MMR genes in ovarian cancer patients. Our results showed that high mRNA levels of MSH6, MLH1, and PMS2 were associated with a favorable OS in ovarian cancer, suggesting these MMR genes may serve as potential positive prognostic indicators in ovarian cancer patients treated with platinum-based chemotherapy. These findings were consistent with previous studies in endometrial cancer and pancreatic cancer. Cohn et al. [16] evaluated four MMR genes, MLH1, MSH2, MSH6, and PMS2, in 336 endometrial cancer samples by immunohistochemistry and found a significantly unfavorable disease-free survival in patients with loss of MLH1 and MSH2 expression compared with normal expression in either protein. In pancreatic cancer, extensive MLH1 expression was significantly associated with favorable differentiation and less lymph node metastasis, and univariate analysis showed that patients with low expression of MLH1 in tumor tissues had significantly poorer overall survival [32]. In further support from ovarian cancer, Mann et al. [20] detected common variants in the MMR pathways, such as MLH1 rs1799977 and MSH3 rs6151662, had negative effect on survival in serous type ovarian cancer patients. Clearly, these data suggested that sufficient MMR system appears to be associated with an improved survival in ovarian cancer. It is likely that the ability to recognize and repair DNA mismatches favors improved cancer outcomes in women with ovarian cancer. Another feasible explanation involves MMR system and its association with chemoresistance. Ding and his colleagues reported MLH1 expression could sensitize ovarian cancer cells to cell death [33]. In their study, the percentage of cells undergoing cisplatin-induced cell killing was higher in MLH1-proficient cells than in MLH1-defective cells [33]. Additionally, PMS2 is required for cisplatin-induced activation of p53, a member of the p53 family of transcription factors with proapoptotic activity [34]. In further support, Jia et al. [35] revealed PMS2 expression in epithelial ovarian cancer is post-translationally regulated by Akt and essential for platinum-induced apoptosis in a recently published paper.

We further investigated the association between MMR genes and OS in different subtype ovarian cancer. Interestingly, our date showed that increased MSH6 and PMS2 mRNA levels were correlated with a favorable OS in serous ovarian cancer, but not in endometrioid ovarian cancer. These results suggest that the correlation between MMR genes and the prognosis of ovarian cancer varies substantially across subtypes.

In addition, our study showed the expression of MSH6, MLH1 and PMS2 gene exerted positive influences on OS of late-stage and poor-differentiated ovarian cancer patients, but not of early stage and well-differentiated ovarian cancer patients. These results further demonstrated the importance of active MMR system in inhibiting the progress of ovarian cancer, which recognize and repair DNA mismatches.

The major strengths of this study were its large sample size as well as the length of the follow-up. However, due to the database only including 30 endometrioid cancer patients, the results may lack reliability. Ovarian cancer includes many different subtypes, we only analyzed serous cancer and endometrial cancer, and the results in the rest subtypes are still unknown. On the other hand, MMR gene mRNA was extracted from cancer tissues, which were composed of many type of cells. Thus, MMR gene mRNA expression in the individual cell types is still unknown and may be different. Therefore, further investigation will be needed to identify the role and clinical significance of the individual MMR genes in different types of cells.

In summary, using the KM plotter database, we systemically investigated the prognostic values of seven MMR genes in ovarian cancer and found increased mRNA expression of MSH6, MLH1 and PMS2 was correlated to an improved OS in 1335 human ovarian cancer patients treated with platinum-based chemotherapy, especially in late-stage and poor-differentiated ovarian cancer. Our data suggests that sufficient MMR system is associated with an improved survival in ovarian cancer. MMR gene may be potential prognosis predictors in ovarian cancer patients. Further studies to validate these results at the in situ protein expression level in human ovarian cancer tissues are warranted.
